# Depression, anxiety, and mental state in patients with early-stage dementia: comparison of reminiscence therapy and cognitive stimulation therapy

**DOI:** 10.1590/1980-5764-DN-2025-0354

**Published:** 2026-02-16

**Authors:** Zeynep Gümüş Demir, Idil Arasan Doğan, Remziye Keskin, Defne Demet Koyutürk

**Affiliations:** 1 Üsküdar University, Faculty of Human and Social Sciences, Department of Psychology, İstanbul, Turkey.; 2 Üsküdar University, Institute of Social Sciences / Psychology PhD, İstanbul, Turkey.; 3 Üsküdar University, Institute of Social Sciences / Applied Psychology, İstanbul, Turkey.; 4 Darülaceze Presidency, Social Worker, İstanbul, Turkey.

**Keywords:** Dementia, Depression, Anxiety, Cognition, Therapeutics, Demência, Depressão, Ansiedade, Cognição, Terapêutica

## Abstract

Psychosocial interventions may differentially target affective and cognitive symptoms in mild dementia, yet comparative evidence in long-term care is limited.

**Objective:**

To compare the effects of Reminiscence Therapy (RT) and Cognitive Stimulation Therapy (CST) on depression, anxiety, and cognitive functioning in older adults with mild dementia living in long-term care facilities.

**Methods:**

A quasi-experimental, quantitative study was conducted with 24 participants diagnosed with mild dementia, allocated equally to RT (n=12) and CST (n=12). Thirteen were male and eleven were female. Data were collected using a Sociodemographic Information Form, the Geriatric Depression Scale, the Beck Anxiety Inventory, and the Standardized Mini-Mental State Examination. Both groups received 10 weekly sessions lasting 90 minutes each.

**Results:**

Distinct patterns emerged between the interventions. RT was more effective for emotional symptoms: depressive symptoms decreased significantly (t(11)=8.26, p<0.001) and anxiety also improved (t(11)=10.41, p<0.001). CST led to greater gains in cognitive performance (t(11)=-2.42, p=0.034) while also reducing depressive (t(11)=3.08, p=0.010) and anxiety symptoms (t(11)=5.05, p<0.001). These differential effects suggest unique therapeutic mechanisms.

**Conclusion:**

Both RT and CST may provide valuable psychosocial benefits for older adults with mild dementia. The findings highlight the need for individualized intervention strategies that consider each person’s emotional and cognitive profiles when designing mental health care plans in long-term care settings.

## RESUMO.

Intervenções psicossociais podem direcionar de forma diferenciada sintomas afetivos e cognitivos na demência leve, porém as evidências comparativas em instituições de longa permanência são limitadas.

**Objetivo:**

Comparar os efeitos da Terapia de Reminiscência (TR) e da Terapia de Estimulação Cognitiva (TEC) sobre depressão, ansiedade e funcionamento cognitivo em idosos com demência leve residentes em instituições de longa permanência.

**Métodos:**

Estudo quantitativo, quase-experimental, realizado com 24 participantes diagnosticados com demência leve, distribuídos igualmente entre TR (n=12) e TEC (n=12). Treze eram do sexo masculino e onze do sexo feminino. Os dados foram coletados por meio de um Formulário de Informações Sociodemográficas, da Escala de Depressão Geriátrica (EDG), do Inventário de Ansiedade de Beck (IAB) e do Mini Exame do Estado Mental (MEEM) padronizado. Ambos os grupos receberam 10 sessões semanais com duração de 90 minutos cada.

**Resultados:**

Padrões distintos emergiram entre as intervenções. A TR mostrou-se mais eficaz para os sintomas emocionais: os sintomas depressivos reduziram significativamente (t(11)=8,26; p<0,001) e a ansiedade também apresentou melhora (t(11)=10,41; p<0,001). A TEC resultou em maiores ganhos no desempenho cognitivo (t(11)=-2,42; p=0,034), além de reduzir sintomas depressivos (t(11)=3,08; p=0,010) e de ansiedade (t(11)=5,05; p<0,001). Esses efeitos diferenciais sugerem mecanismos terapêuticos específicos.

**Conclusão:**

Tanto a TR quanto a TEC podem oferecer benefícios psicossociais relevantes para idosos com demência leve. Os achados ressaltam a necessidade de estratégias de intervenção individualizadas, que considerem o perfil emocional e cognitivo de cada pessoa ao planejar cuidados em saúde mental em instituições de longa permanência.

## INTRODUCTION

While the aged population is increasing rapidly worldwide, Turkey has announced its aged population as 10.2% - in double digits for the first time - by 2023. This situation indicates that age-related problems will increase rapidly. Old age is a period in which individuals face social, psychological, and physical changes, in addition to biological aging, making adaptation to these changes essential. During this process, individuals often evaluate their life experiences and review their past. Physical and mental decline is common during this period, and changes in cognitive functioning are frequently associated with dementia syndrome. Dementia is the most common neurodegenerative disease diagnosed in older adults due to increased life expectancy. Forgetfulness, impairments in activities of daily living and intellectual functions, and accompanying psychological symptoms are commonly observed during this process^1^. It is known that more than 55 million people worldwide are affected by dementia, which gradually causes dependence on others for daily life activities. This number is estimated to reach 139 million by the 2050s^2^ ^,^ ^3^. Given the impact of dementia on mental health, the importance of innovative and value-adding approaches in caregiving processes for older adults is considerable^4^. Recent studies on the prevalence of dementia types in Turkey indicate that Alzheimer disease (AD) is the most common type of dementia. A study conducted in 2023 found the prevalence of dementia in Turkey to be 11.1%, with rates increasing with age. AD accounts for 60-70% of all dementia cases, followed by vascular dementia as the second most common type^5^. Globally, one in three older adults dies from AD or dementia, while only four countries have established national plans to support patients with dementia and their caregivers. The caregiving process for dementia, which is both progressive and regressive, becomes a major problem if not managed appropriately^6^. Dementia usually begins as mild cognitive impairment and progresses through three stages. It is known that dementia, which requires long-term care, negatively affects social life and activities of daily living, while also increasing symptoms of anxiety and depression^7^. Difficulties in coping with these challenges and the need for professional support often make institutional care necessary^8^. Regulating the environment and implementing positive interventions by anticipating the psychological needs of older adults with dementia receiving institutional care are critical to disease management^9^. This approach is important for ensuring quality care, increasing participation in social activities, and reducing psychiatric symptoms, therevy making the process more manageable^10^. In institutional care, organizing activities to help individuals with dementia maintain functionality and a sense of usefulness supports coping with losses while preserving self-esteem. This, in turn, promotes greater stability in daily life. Moreover, incorporating therapeutic approaches into caregiving processes is highly valuable for both individuals with dementia and their caregivers^11^. Reminiscence therapy (RT) and cognitive stimulation therapy (CST) are considered the most beneficial therapeutic methods in institutional settings. RT is a model frequently used in day care centers, nursing homes, and residential facilities. CST aims to enhance the cognitive functions of individuals with dementia and support their social interactions. However, the success of these therapies depends on the individual’s personal characteristics, the stage of the disease and the competence of the practitioner. Inadequate training or experience among practitioners may result in suboptimal outcomes or even stress among participants^12^. Psychoeducation programs for institutional staff and interventions led by experienced psychologists and social workers are therefore essential. This study, which applied RT and CST comparatively, was carried out with older adults with mild-stage dementia residing in a nursing home. The aim was to produce a positive change in their levels of depression, anxiety, and overall mental state.

## METHODS

This study followed a quasi-experimental design. Participants were not randomly assigned; rather, they were allocated to two intervention groups (RT and CST) within the natural institutional setting. Pre-test and post-test assessments were conducted in both groups to evaluate the effects of the interventions. Therefore, the design is described as quasi-experimental, as it lacked randomization and a control group typically present in clinical trials. A total of 24 older adults with a clinical diagnosis of mild dementia residing in a long-term care facility in Istanbul, Turkey, were included in the study. Participants were assigned to either the RT group or the CST group using matched assignment based on gender and age to ensure comparability. The non-random allocation reflected the real-life clinical context and institutional constraints.

### Intervention

(RT is a psychosocial intervention that encourages older adults to recall and share past experiences in structured sessions, aiming to improve emotional well-being and social interaction. Its effectiveness in dementia care has been demonstrated in Cochrane reviews^13^ ^,^ ^14^ and subsequent systematic reviews^15^. CST is a structured, group-based program designed to enhance cognitive functioning in individuals with dementia, particularly in mild-to-moderate stages^16^ ^,^ ^17^. Both approaches are recommended as evidence-based interventions in international dementia care guidelines. RT typically involves the use of prompts such as photographs, music, or objects to stimulate memory, whereas CST sessions include themed activities designed to actively engage multiple cognitive domains.

### Ethics statement

The study was approved by the Üsküdar University Ethics Committee (reference 61351342/KASIM2023-26). Before obtaining informed consent, the study purpose, methodology, and procedures were explained in detail.

Informed consent was obtained from all participants.

### Data collection instruments

Pre- and post-intervention data were collected using standardized instruments:

• Geriatric Depression Scale (GDS): Developed by Burke et al. and validated in Turkish by Durmaz et al.^18^;
• Beck Anxiety Inventory (BAI): Originally developed by Beck et al. and adapted into Turkish by Ulusoy et al., with a Cronbach’s alpha of 0.93^19^ ^,^ ^20^;
• Standardized Mini-Mental Test (SMMT): Adapted by Güngen et al. from the original version by Folstein et al., including a form suitable for illiterate individuals; Turkish version reliability was α=0.84^21^ ^,^ ^22^.

Note on Sociodemographic Data: Participant characteristics such as age, gender, education, and health history were collected using a structured form and summarized descriptively to maintain focus on therapeutic outcomes.

## INTERVENTION PROGRAM

The interventions consisted of a 10-week structured program of RT and CST. Sessions were conducted once per week, lasting 90 minutes each. Details of both programs are provided below. The content and structure of both therapeutic programs were systematically planned according to the principles of each approach. Weekly session topics for the RT and CST programs are summarized in Table 1 and Table 2, respectively.

**Table 1.** Session structure and topics of the Reminiscence Therapy program.

Reminiscence Therapy Program	Subject
Session 1	• Welcome and introduction of the session host • Explanation of group rules • Self-awareness, awareness of others, and identification of goals and process • Self-introduction of each participant, with support if needed • Sharing names, nicknames (if any), place and date of birth, occupations, and hobbies • Summarizing activities and discussing group interactions
Session 2	• Welcome and introduction of the session host • Reminder of group rules • Reviewing what was discussed in the previous week • Promoting hopefulness and life review • Recalling school memories; for those who cannot recall, supporting recall through group prompts • Summarizing activities and discussing interactions
Session 3	• Welcome and introduction of the session host • Reminder of group rules • Reviewing what was discussed in the previous week • Recalling childhood games, with supportive cues for those who have difficulty remembering • Summarizing activities and discussing interactions • Discussing the place and conditions in which participants live, comparing past and present
Session 4	• Welcome and introduction of the session host • Reviewing what was discussed in the previous week • Discussing national and religious holidays; using related photographs as visual aids • Supporting personal memory within this context • Summarizing activities and discussing interactions
Session 5	• Welcome and introduction of the session host • Reviewing what was discussed in the previous week • Discussing relationships and marriage • Married participants sharing memories from their wedding ceremonies • Participants who are single may share memories of past relationships or personal reflections on the topic • Summarizing activities and discussing interactions
Session 6	• Welcome and introduction of the session host • Reviewing what was discussed in the previous week • Discussing an object participants carry with them or particularly like. • Summarizing activities and discussing interactions • Announcement to bring photographs to the next session
Session 7	• Welcome and introduction of the session host • Reviewing what was discussed in the previous week • Displaying and discussing the photograph each participant brought, including the person in the photo and the associated memory • Discussing meaningful life events and their personal impact • Summarizing activities and discussing interactions
Session 8	• Welcome and introduction of the session host • Reviewing what was discussed in the previous week • Sharing favorite music and artists; listening to the selected songs and, if possible, singing together • Discussing the artist and the significance of the song • Summarizing activities and discussing interactions
Session 9	• Welcome and introduction of the session host • Reviewing what was discussed in the previous week • Discussing family life and relationships with family members • Using the “Circle” technique from the Interpersonal Therapeutic Approach during discussion • Summarizing activities and discussing interactions • Discussing materials needed for the closing party the following week
Session 10	• Welcome and introduction of the session host • Reviewing what was discussed in the previous week • Organizing a closing party using the materials available • Sharing memories of simultaneous or individual celebrations • Reviewing what was completed throughout the ten-week program • Closing remarks and final announcement

**Table 2.** Session structure and topics of the Cognitive Stimulation Therapy program.

Cognitive Stimulation Therapy Program	Subject
Session 1	• Welcome and introduction of the session host • Explanation of group rules • The practitioner writes the date on the board (day, month, year). • Writing the season and weather on the board • Generating name options for the group and reaching a consensus • Distributing a sheet with flowers drawn on it and asking participants to color according to their emotions. • Discussing the meaning of the chosen colors • Determining a group song and singing it together • Group photograph session
Session 2	• Welcome • Joint reminder of group rules • The practitioner writes the date on the board (day, month, year). • Writing the season and weather on the board • Using a cloth ball, participants describe themselves it is thrown to them. • Singing the group song together • Group photograph session
Session 3	• Welcome • Joint reminder of group rules • The practitioner writes the date on the board (day, month, year). • Writing the season and weather on the board • Using a cloth ball, participants describe happy and unhappy times when he ball is thrown to them. • Throwing the ball into a basket and recalling each scored number • Singing the group song together • Group photograph session
Session 4	• Welcome • Joint reminder of group rules • The practitioner writes the date on the board (day, month, year). • Writing the season and weather on the board • Distributing papers for writing memories of childhood and youth; group discussion afterward • Drawing the environment/room where participants lived while growing up and discussing the drawings • Singing the group song together • Group photograph session
Session 5	• Welcome • Joint reminder of group rules • The practitioner writes the date on the board (day, month, year). • Writing the season and weather on the board • Preparing a dinner menu from items placed on the table • Each participant describes their experience in detail • Singing the group song together • Group photograph session
Session 6	• Welcome • Joint reminder of group rules • The practitioner writes the date on the board (day, month, year). • Writing the season and weather on the board • Discussing a written narrative by collecting input from each participant • Discussing gender roles • Discussing behaviors of younger and older generations • Planting plants and assigning each participant responsibility for monitoring growth, recording the planting date • Singing the group song together • Group photograph session
Session 7	• Welcome • Joint reminder of group rules • The practitioner writes the date on the board (day, month, year). • Writing the season and weather on the board • Analyzing and discussing photographs of notable individuals, historical sites, and landscapes • Each participant describes details in the photographs (*e.g.*, similarities, differences, colors, theme, etc.) • Participants draw pictures of places they have previously visited before and discuss the memories. • Singing the group song together • Group photograph session
Session 8	• Welcome • Joint reminder of group rules • The practitioner writes the date on the board (day, month, year). • Writing the season and weather on the board • Writing incomplete sentences and asking participants to complete them • Sharing experiences regarding what made them happy or unhappy during the week • Singing the group song together • Group photograph session
Session 9	• Welcome • Joint reminder of group rules • The practitioner writes the date on the board (day, month, year). • Writing the season and weather on the board • Finding and placing appropriate shapes on the floor • Working with clay dough to create figures based on model images; labeling the work with the creator’s name and planning to bring it in the next session • Announcement to bring the grown plant to the next session • Singing the group song together • Group photograph session
Session 10	• Welcome • Joint reminder of group rules • The practitioner writes the date on the board (day, month, year). • Writing the season and weather on the board • Group discussion reviewing activities carried out across all sessions, written on the board • Reviewing the grown plant • Displaying the clay figures on a table • Digital sharing of the photo album created across sessions; printed albums are distributed to participants • Singing the group song together • Ending the program with closing remarks and well-wishes

### Data analysis

Before beginning data analysis, the collected data were formatted and transferred to the Statistical Package for the Social Sciences (SPSS), version 25. In the next stage, kurtosis and skewness coefficients were examined to evaluate the assumption of normal distribution. Values between -3 and +3 for both coefficients indicated that the assumption of normal distribution was met.

### Findings

The study sample consisted of two equal groups (n=12 each) receiving either RT or CST. In the RT group, participants were evenly distributed by gender, and most had at least basic education, with 25% having university-level education. A small proportion had a psychiatric history (8.3%), none was using psychiatric medication, and all had at last one physical illness. The mean age was 76.17 years (SD=7.67), ranging from 66 to 93 years. In the CST group, most participants were male, and the majority had primary education. While 16.7% had a psychiatric history, only one individual was using psychiatric medication. Physical illness was reported in 91.7% of participants. The mean age was 77.00 years (SD=6.92), with an age rage of 68 to 93 years.

When GDS (pre-test), SMMT (pre-test), and BAI (pre-test) scores were compared between the RT and the CST groups, no significant difference was found (p>0.05) (Table 3).

**Table 3.** Comparison of Geriatric Depression Scale (pre-test), Standardized Mini-Mental Test (pre-test), and Beck Anxiety Inventory (pre-test) scores between the Reminiscence Therapy Group and the Cognitive Stimulation Therapy Group.

Dependent Variables	Reminiscence therapy group (n=12)		Cognitive stimulation therapy group (n=12)		*t*	p-value
	$\bar{X}$	*SS*	$\bar{X}$	*SS*		
Geriatric Depression Scale (pre-test)	6.75	2.14	8.25	2.90	-1.44	0.163
Standardized Mini-Mental Test (pre-test)	20.33	2.19	19.75	1.96	0.69	0.499
Beck Anxiety Inventory (pre-test)	14.00	2.98	14.33	3.47	-0.25	0.803

Notes: ***p<0.001; **p<0.01; *p<0.05. Test Used: Independent Samples t-Test.

When the SMMT scores were compared between pre-test and post-test, no significant difference was observed between the groups (p>0.05).

When GDS scores were compared between pre-test and post-test, a significant difference was observed (t(11)=8.26, p<0.001). The mean pre-test score (6.75±2.14) was significantly higher than the mean post-test scores (4.17±1.59) (Table 4).

**Table 4.** Comparison of Geriatric Depression Scale, Standardized Mini-Mental Test, and Beck Anxiety Inventory Pre-Test and Post-Test Scores in the Reminiscence Therapy Group.

Dependent Variables	Pre-test (n=12)		Post-test (n=12)		*t*	p-value
	$\bar{X}$	*SS*	$\bar{X}$	*SS*		
Geriatric Depression Scale	6.75	2.14	4.17	1.59	8.26	<0.001***
Standardized Mini-Mental Test	20.33	2.19	20.50	2.24	-1.48	0.166
Beck Anxiety Inventory	14.00	2.98	6.00	1.35	10.41	<0.001***

Notes: ***p<0.001; **p<0.01; *p<0.05. Test Used: Dependent Samples t-Test.

When BAI scores were compared between pre-test and post-test, a significant difference was found (t(11)=10.41, p<0.001). The mean pre-test score (14.00±2.98) was significantly higher than the mean post-test score (6.00±1.35) (Table 4).

When the GDS scores in the CST group were compared between pre-test and post-test, a significant difference was observed (t(11)=3.08, p<0.01). The mean pre-test score (8.25±2.90) was significantly higher than the mean post-test score (6.00±2.30) (Table 5).

**Table 5.** Comparison of Geriatric Depression Scale, Standardized Mini-Mental Test, and Beck Anxiety Inventory Pre-Test and Post-Test Scores of Cognitive Stimulation Therapy Group.

Dependent variables	Pre-test (n=12)		Post-test (n=12)		*t*	p-value
	$\bar{X}$	*SS*	$\bar{X}$	*SS*		
Geriatric Depression Scale	8.25	2.90	6.00	2.30	3.08	0.010**
Standardized Mini-Mental Test	19.75	1.96	20.58	1.78	-2.42	0.034*
Beck Anxiety Inventory	14.33	3.47	8.42	2.75	5.05	<0.001***

Notes: ***p<0.001; **p<0.01; *p<0.05. Test Used: Dependent Samples t-Test.

When the SMMT scores were compared between pre-test and post-test in this group, a significant difference was found (t(11)=-2.42, p<0.05). The mean pre-test score (19.75±1.96) was significantly lower than the mean post-test scores (20.58±1.78) (Table 5).

When BAI scores were compared between pre-test and post-test, a significant difference was found (t(11)=5.05, p<0.001). The mean pre-test score (14.33±3.47) was significantly higher than the mean post-test score (8.42±2.75) (Table 5).

When the SMMT (post-test) scores were compared between the RT and CST groups, no significant difference was observed (p>0.05) (Table 6).

**Table 6.** Comparison of Geriatric Depression Scale (post-test), Standardized Mini-Mental Test (post-test), and Beck Anxiety Inventory (post-test) Scores between the Reminiscence Therapy Group and the Cognitive Stimulation Therapy Group.

Dependent Variables	Reminiscence therapy group (n=12)		Cognitive stimulation therapy group (n=12)		*t*	p-value
	$\bar{X}$	*SS*	$\bar{X}$	*SS*		
Geriatric Depression Scale (post-test)	4.17	1.59	6.00	2.30	-2.28	0.033*
Standardized Mini-Mental Test (post-test)	20.50	2.24	20.58	1.78	-0.10	0.920
Beck Anxiety Inventory (post-test)	6.00	1.35	8.42	2.75	-2.74	0.012*

Notes: ***p<0.001; **p<0.01; *p<0.05. Test Used: Independent Samples t-Test.

When the GDS (post-test) scores were compared, a significant difference was found between the groups (t(22)=-2.28, p<0.05). The CST group mean score (6.00±2.30) was significantly higher than the RT group mean score (4.17±1.59) (Table 6).

When BAI (post-test) scores were compared, a significant difference was found (t(22)=-2.74, p<0.05). The CST group mean score (8.42±2.75) was significantly higher than the RT group mean score (6.00±1.35) (Table 6).

## DISCUSSION

This study was conducted with older adults diagnosed with mild-stage dementia in a long-term care home. The effects of RT and CST on mental state, anxiety and depression levels were examined. In the literature, the positive effects of RT on depression and anxiety in older adults^23^ ^,^ ^24^, as well as the potential of CST to slow cognitive decline^25^, make comparison of these two therapies meaningful.

As a result of the study, depression, anxiety, and mental status of older adults with mild dementia who participated in RT and CST improved. While both therapies led to significant improvements in depression and anxiety symptoms, RT resulted in a greater reduction in emotional symptoms, whereas CST showed more improvement in cognitive functioning. Previous literature has reported that placement of older adults in long-term institutional care can lead to intense stress, loneliness, anxiety, and depression. Other problems identified in institutionalized care include difficulty establishing meaningful interpersonal relationships with residents and staff, a sense of lost identity, hopelessness, sadness, boredom and lack of social support^26^. In addition to these experiences, it is also known that dementia-related cognitive decline and psychological symptoms may interact and intensify during the time spent in institutional settings^27^. Among individuals diagnosed with dementia, gradually decreasing cognitive functions may aggravate neuropsychiatric symptoms^28^. With the increasing need for caregiving, caregiving itself may become a burden^25^. In this context, functional and supportive psychotherapeutic interventions are increasingly important. Various studies on RT have shown positive outcomes in mood, well-being, anxiety, engagement, and quality of life among older adults^18^ ^,^ ^29^. RT is recommended as part of routine care for individuals with dementia. CST is based on the “use it or lose it” principle, whereby stimulation of different mental abilities may slow cognitive decline through neuronal activation and improved function. CST is a brief, non-pharmacological psychological intervention for individuals with mild to moderate dementia^27^. It is typically implemented in group settings and haa been shown to provide mental stimulation through structured activities and discussions. Group CST is recognized as an evidence-based psychosocial intervention for people living with dementia^30^, with substantial evidence supporting its short- and long-term benefits^31^. The findings of this study indicated that older adults who participated in both RT and CST groups experienced reductions in depression levels. In support of these results, many studies have reported decreases in depression among individuals who received RT^32^ ^,^ ^33^. However, some studies have reported no significant change in depression following RT. Numerous studies indicate that CST has a beneficial effect on depression in older adults with dementia^34^ ^,^ ^35^. Another result of this study I that anxiety levels decreased among participants who engaged in both therapies. RT is considered an approach with strengths that support person-centered care because it makes use of past memories in individuals’ lives (memories that are often thought to be preserved in dementia). In the literature, several studies show that RT^36^ ^,^ ^37^ ^,^ ^38^ ^,^ ^39^ and CST^40^ ^,^ ^41^ reduce anxiety, supporting the findings obtained in the present study^42^. Among the research findings is the observation of improvement in the cognitive functions of patients with mild-stage dementia who received CST. Many studies have reported similar results^43^. RT has gained attention in recent years for its role in stimulating cognitive functions in older adults. In addition, RT is considered a useful and feasible intervention for individuals with AD and for coping with mental health problems^44^. In the current study, no significant difference was found between groups regarding cognitive functions among older adults diagnosed with mild-stage dementia following the intervention. In contrast to the present findings, there are studies demonstrating that RT supports improvement in cognitive functions^45^ ^,^ ^46^. In their research, Li and colleagues (2020) suggest that RT has a beneficial effect on depressive and neuropsychiatric symptoms in patients diagnosed with AD^47^. In a multicenter randomized controlled trial examining the effects of RT on depression, quality of life, and cognition in older adults with AD and vascular dementia, it was emphasized that the therapy may have beneficial effects on cognition and quality of life^48^. Thomas and Sezgin^49^ reached similar conclusions; in a systematic review and meta-analysis of dementia patients living in long-term care facilities, they suggest that RT may be effective in reducing depression and improving quality of life and cognition. When comparing RT and CST, the depression and anxiety levels of participants in the CST group were found to be significantly higher than those in the RT group. While no difference in mental status was observed in the RT group, a statistically significant difference was found in the mental status levels of older adults who participated in CST. This study has limitations, including its single-institution setting, regional sample, small group size, and lack of post-intervention follow-up, which limit generalizability. Future research in more diverse settings is needed. Adapting therapies to cultural contexts may enhance their impact. These findings support the integration of RT and CST into routine care for individuals with dementia. Policymakers and healthcare providers should promote these evidence-based interventions and ensure staff training, improving cognitive and emotional outcomes and enhancing quality of care in long-term care institutions.

### Limitations

Among the limitations of this study, the foremost is the relatively small sample size. Conducting the research with only 24 participants restricts statistical power, particularly for subjective outcome measures such as depressive

and anxiety symptoms. Furthermore, the single-institution design limits the generalizability of the findings. Replication with larger and multicenter samples will be crucial for confirming these results in future research.

Secondly, although the results are largely consistent with previous literature, the novelty of this study lies in its cultural context. All participants were older adults residing in long-term care facilities in Turkey. Since most existing evidence comes from Western societies, the findings contribute to validating these therapeutic approaches in a different cultural setting and highlighting the importance of culturally sensitive psychosocial interventions. In addition, the limited sample size reduces the statistical power of the results; future studies should employ further analyses, such as effect sizes (*e.g*., Cohen’s d), correlation coefficients, or regression models.

In conclusion, this study demonstrates that RT can effectively alleviate emotional symptoms, while CST supports cognitive functioning in older adults with mild dementia. The findings emphasize the importance of tailoring care plans to individual needs by integrating both approaches.

## DATA AVAILABILITY STATEMENT

The data that support the findings of this study are available from the corresponding author upon reasonable request. Due to privacy and ethical restrictions, individual participant data cannot be shared publicly.
